# The importance of proteins of the RNase II/RNB-family in pathogenic bacteria

**DOI:** 10.3389/fcimb.2014.00068

**Published:** 2014-06-03

**Authors:** Rute G. Matos, Cátia Bárria, Ricardo N. Moreira, Susana Barahona, Susana Domingues, Cecília M. Arraiano

**Affiliations:** Control of Gene Expression Laboratory, Instituto de Tecnologia Química e Biológica, Universidade Nova de LisboaOeiras, Portugal

**Keywords:** RNase R, virulence factors, Ribonucleases, RNA degradation, trans-translation, RNA processing, post-transcriptional regulation, RNase II

Ribonucleases (RNases) are important factors for the establishment of virulence in an increasing number of pathogens. These enzymes are responsible for the maturation and degradation of RNA molecules, being key players in RNA metabolism. In this opinion we will focus on exoribonucleases from the RNB family, whose members are known to be involved in the virulence of several microorganisms.

Members of this family are present in all domains of life. They hydrolyze RNA in the 3'–5' direction in a processive way (reviewed in Matos et al., 2011). RNase II from *Escherichia coli* is the prototype of this family, which also includes bacterial RNase R and eukaryotic Rrp44/Dis3 proteins. These proteins present a similar modular organization with cold shock domains (CSD) at the N-terminal region involved in RNA binding, a central RNB domain responsible for catalysis, and a C-terminal S1 domain, also involved in RNA binding (Amblar et al., 2006; Frazão et al., 2006). The RNB domain (characteristic from this family of enzymes) is very well-conserved, and the residues involved in catalysis are found in all members of the family (Barbas et al., 2008). These results point toward a conservation of the mechanism of action, as shown by the characterization of several enzymes of this family (Matos et al., 2009; Reis et al., 2013a).

Exoribonucleases from the RNB-family have a wide spectrum of functions in the cell. In eukaryotes Rrp44/Dis3 and Dis3L1 are the catalytic subunit of the exosome, a crucial RNA degradation complex (reviewed in Arraiano et al., 2013). Dis3L2 was recently discovered and defines a novel eukaryotic RNA degradation pathway (Malecki et al., 2013). In prokaryotes, they are often essential for growth and viability and can be developmentally regulated. They are important for stress responses and are also involved in RNA and protein quality control (reviewed in Matos et al., 2011). We will discuss some of these functions, focusing on RNase R, which has been implicated in pathogenesis.

## RNase R levels change according to the environment

RNase II and RNase R were shown to be modulated by the environment.

Under stress conditions, namely stationary phase and cold shock, RNase R levels increase in the cell, suggesting a role for the protein under these conditions (Cairrão et al., 2003; Chen and Deutscher, 2005; Andrade et al., 2006; Moreira et al., 2012). In some pathogenic organisms, such as *Aeromonas hydrophila, Legionella pneumophila*, and *Pseudomonas syringae*, RNase R is even crucial for cell survival at low temperatures (Purusharth et al., 2007; Charpentier et al., 2008; Erova et al., 2008). Adaptation to cold implies a rearrangement in the cell metabolism. In this situation, most of the protein production stops (reviewed by Barria et al., 2013), and RNase R is one of the few exceptions, being induced several fold (Cairrão et al., 2003; Andrade et al., 2006; Moreira et al., 2012). RNase R is able to cleave secondary structures, a role that may be extremely important at low temperatures due to the stabilization of structured RNA. This enzyme is the only exoribonuclease which is able to digest highly structured RNA without the help of a helicase (reviewed in Matos et al., 2011). It was also shown that *rnr* mRNA levels are lower in stationary phase cultures but the level of the protein is higher. Liang and Deutscher have shown that RNase R is also regulated at the level of protein stability. In exponential phase, acetylation targets RNase R for degradation in a tmRNA/SmpB dependent way. On the contrary, in stationary phase the protein that acetylates RNase R is absent and thus, RNase R is stabilized (Liang and Deutscher, 2010, 2012). RNase II, the other protein of this family present in *E. coli*, was also shown to be controlled post-translationally and in strains without *gmr* (gene modulating RNase II), the protein is stabilized (Cairrão et al., 2001).

## RNase R, ribosomes, and trans-translation

RNase R interacts with ribosomes (Liang and Deutscher, 2013; Malecki et al., 2014), mainly with the ribosomal 30S subunit (Malecki et al., 2014). It was also shown to interact with the ribosomal protein S12 (Ge et al., 2010; Liang and Deutscher, 2013; Strader et al., 2013). Together with the YbeY nuclease, RNase R is able to efficiently cleave defective ribosomes *in vitro* (Jacob et al., 2013). The quality control of ribosomes is critical to ensure proper protein translation. RNase R, in concert with the exoribonuclease PNPase, was shown to be involved in rRNA quality control (Cheng and Deutscher, 2003).

*Trans*-translation is an elegant surveillance pathway that directs deficient proteins and mRNAs for degradation while rescuing stalled ribosomes. During *trans*-translation an RNA called transfer-messenger RNA (tmRNA), in association with a small RNA-binding protein (SmpB), recognizes stalled ribosomes on defective mRNAs. tmRNA functions both as tRNA and as mRNA, and contains a specialized open reading frame that encodes a peptide tag. tmRNA is loaded onto the defective RNA and the stalled ribosome switches from the translation of the defective mRNA to the translation of the mRNA domain of tmRNA. After translating the tmRNA reading frame, the stalled ribosome is released and the protein carries a C-terminal tag that is recognized and degraded by the cellular proteases (reviewed in Karzai et al., 2000, and more recently in Barends et al., 2011; Keiler and Ramadoss, 2011). *Trans*-translation has also been associated with deficiencies in stress-response mechanisms and pathogenicity (Keiler, 2007), and it is often required when bacteria alter their genetic program in response to these adverse situations (Keiler, 2008).

One of the first indications for the involvement of *E. coli* RNase R in the quality control of proteins and RNAs was its association with a ribonucleoprotein complex containing tmRNA and SmpB (Karzai and Sauer, 2001). *E. coli* RNase R was subsequently shown to be required for the maturation of tmRNA under cold-shock (Cairrão et al., 2003). In *P. syringae* and *Caulobacter crescentus*, degradation of tmRNA was also demonstrated to be dependent on RNase R (Hong et al., 2005; Purusharth et al., 2007). After ribosomal release, faulty mRNAs must be rapidly degraded to avoid their engagement in a new translation event. RNase R was further shown to be the key exoribonuclease involved in the selective degradation of non-stop mRNAs (Richards et al., 2006). The Lysine-rich stretch at the C-terminus of RNase R is essential for the enzyme's recruitment to the ribosomes that are stalled and for its activity on the degradation of defective transcripts (Ge et al., 2010). The interaction between RNase R and the ribosomes was shown to depend on both functional SmpB and tmRNA, and is increased by overexpression of a non-stop mRNA (Ge et al., 2010; Liang and Deutscher, 2013). A proper engagement of RNase R seems to be determinant for the RNase R role in *trans*-translation (Ge et al., 2010). Binding to ribosomes stabilizes RNase R and controls its activity by sequestering it away from many functional RNAs (Liang and Deutscher, 2013). RNase R stability is also modulated by tmRNA/SmpB, whose interaction with RNase R ultimately targets the enzyme for proteolytic degradation (Liang and Deutscher, 2010). Binding of the tmRNA/SmpB complex to the RNase R C-terminal region is in turn dependent on previous RNase R acetylation (Liang et al., 2011).

The relation between RNase R and the *trans*-translation components has recently been strengthened by the observation that RNase R and SmpB are co-transcribed and cross-regulated in *Streptococcus pneumoniae* (Moreira et al., 2012). In this bacterium not only RNase R levels are modulated by SmpB, but also SmpB mRNA and protein levels are under the control of RNase R (Moreira et al., 2012).

## RNase R in pathogenic organisms

Although RNase R has been extensively studied in *E. coli*, homologs have been identified in a wide range of species. This enzyme has been implicated in the virulence mechanisms of a growing number of pathogens. *Shigella flexneri* is the causative agent of dysentery. It contains an RNase R homolog that is extremely important for the expression of a variety of invasion factors, such as, IpaB, IpaC, IpaD, and VirG (Tobe et al., 1992). RNase R is the only hydrolytic exoribonuclease present in *L. pneumophila*, a facultative intracellular pathogen. This protein was found to have a significant impact on viability at low temperatures and a role on competence induction of this bacterium (Charpentier et al., 2008). In *A. hydrophila*, the absence of RNase R negatively influences the motility and attenuates virulence in mice. Additionally, RNase R is induced in cold-shock conditions and is required for survival of *A. hydrophila* in these conditions (Erova et al., 2008). In *P. syringae*, RNase R substitutes PNPase in the RNA degradosome, a multiprotein complex involved in the degradation of mRNA (Purusharth et al., 2005). Even though a direct effect of this protein in *Pseudomonas* spp. virulence is not yet known, a mutant of RNase R shows growth impairment at low temperatures (Purusharth et al., 2007), revealing an important role of this enzyme in cold conditions. In *Mycoplasma genitalium*, RNase R is the only exoribonuclease present and is essential for cell survival. It degrades several substrates including rRNA but it was shown to be sensitive to certain RNA structural features and RNA ribose methylation (Hutchison et al., 1999; Lalonde et al., 2007). In this organism, RNase R, besides its degradative functions, is also involved in RNA processing, namely tRNA maturation (Lalonde et al., 2007; Alluri and Li, 2012). An exception for the virulence associated with this ribonuclease, is the specific case of *Brucella abortus*. In this bacterium, it was observed that a strain lacking RNase R has no impairment with regard to its virulence (Miyoshi et al., 2007).

In the foodborne pathogen *Campylobacter jejuni*, RNase R was shown to be active in a wide range of conditions. The plasticity exhibited by this enzyme may be crucial for the adaptation of *C. jejuni* during the infection process. Moreover, *C. jejuni* RNase R was shown to be involved in adhesion and invasion to eukaryotic cells (Haddad and Arraiano, unpublished).

Taken together, these observations indicate that bacterial strains lacking RNase R exhibit an attenuated virulence phenotype when compared to their parental strains. This is probably connected to the fact that RNase R is a key player in RNA metabolism. Considering the important functions that RNase R has in the establishment of virulence, it can be a potential target to design compounds able to kill specific microorganisms or to reduce their virulence ability. Further studies regarding RNase R function in the control of pathogenesis will certainly help in the comprehension of the RNA-related processes involved in infection.

## Final considerations

Enzymes from the RNB family play crucial roles in the cell, such as RNA processing, turnover, and surveillance. In prokaryotes, several works have highlighted the importance of these proteins, namely RNase R, for the establishment of virulence in some important pathogens. The mechanism by which RNase R is involved in virulence is not yet well-understood. Probably, it has an indirect role by controlling the stability of crucial transcripts that are directly involved in virulence. Moreover, besides its role in RNA metabolism, RNase R has been involved in different cellular processes, such as association with the ribosomes and *trans*-translation, which emphasizes its importance in pathogenesis. We believe that future work will reveal more about the mechanism of action of these proteins and how they affect the infection process. Moreover, recent publications have associated RNB family members with several important diseases (reviewed in Reis et al., 2013b). Although there is an extensive work performed with members of this family of enzymes, it looks like many surprises are yet to come.

### Conflict of interest statement

The authors declare that the research was conducted in the absence of any commercial or financial relationships that could be construed as a potential conflict of interest.
